# Studies on the Role of circRNAs in Osteoarthritis

**DOI:** 10.1155/2021/8231414

**Published:** 2021-09-04

**Authors:** Wei Wu, Jun Zou

**Affiliations:** School of Kinesiology, Shanghai University of Sport, Shanghai, China 200438

## Abstract

**Objective:**

Provide a reference to elucidate the mechanism of circRNAs regulating osteoarthritis (OA) and the clinical treatment.

**Methods:**

Herein, articles about circRNAs (hsa-circ) and osteoarthritis in the recent 5 years have been reviewed and the differential expression and regulatory effect of circRNAs in OA deduced. Based on these conclusions and Protein-Protein Interaction (PPI), Gene Ontology (GO), and Kyoto Encyclopedia of Genes and Genomes (KEGG) analyses of the acquired circRNAs, the potential functions and interactions of circRNAs in OA and the involved signaling pathways are discussed.

**Results:**

A total of 33 studies meeting the inclusion criteria were included in this study, and 27 circRNAs were upregulated and 8 circRNAs were downregulated in OA. A total of 31 circRNAs were finally included in the PPI, GO, and KEGG analyses. From PPI, 12 map nodes and 7 map edges were interrelated. VWF had the biggest node and edge size. From GO, VWF showed a majority of the functions. From KEGG, circRNAs are enriched in PI3K/AKT, human papillomavirus infection (HPI), and focal adhesion (FA) pathways, and VWF was involved in major pathways.

**Conclusion:**

We found that most articles about circRNAs regulating OA in the recent 5 years focused on the mechanism, especially the absorption effect of circ-miRNA as sponges in the recent 2 years, while most of the articles about their functions addressed ECM and PI3K, AKT, and mTOR signaling pathways. Future studies might focus on the functions of circRNAs, and circRNA VWF, with preferable functions, interactions, and involvement, can be used as a biological indicator to detect OA in clinical practice.

## 1. Introduction

Osteoarthritis (OA) is a common clinical disease that has a long process from early inflammation in the joint to the wear and tear of the cartilage layer and the formation of subchondral osteophytes, eventually leading to the failure of the joint to carry out daily movements and perform daily functions [1, 2]. In clinical practice, OA can only be relieved and improved but cannot be cured fully [3, 4]. The mechanism of OA has not yet been defined in existing studies; however, some studies have shown that circular RNAs (circRNAs) play a role in the occurrence and development of OA, but the functions and mechanism of circRNAs in OA were still not very clear [5, 6]. The present study reviewed the articles about circRNAs and OA in the recent 5 years to provide some reference to elucidate the mechanism of circRNAs in OA. Also, based on PPI, GO, and KEGG analysis of the acquired circRNAs, the potential functions of circRNAs in OA and the involved signaling pathways are also discussed in this article. This review can provide some reference for the fundamental research of the prevention and treatment of OA.

## 2. Material and Methods

### 2.1. Data Source

“Circular RNA (circRNA)” and “osteoarthritis (OA)” were used as keywords to search relevant articles from January 1, 2016, to 2021 in China National Knowledge Infrastructure (CNKI), PubMed, and Web of Science. There are no ethics committee approval and informed consent in this article.

### 2.2. Criteria

#### 2.2.1. Inclusion Criteria

Inclusion criteria are as follows: (1) experimental articles with the keywords in the databases and (2) articles with the circRNA ID starting with hsa (human gene).

#### 2.2.2. Exclusion Criteria

Exclusion criteria are as follows: (1) overviews in the databases, (2) repeated articles, (3) articles of poor quality, (4) articles with the circRNA ID starting with mmu (mouse gene), and (5) articles about rheumatoid arthritis (RA).

### 2.3. PPI, GO, and KEGG Analyses

Included circRNAs were retrieved from circBase (http://www.circbase.org/) to check the accuracy of information, and the circRNAs that had no relevant information were deleted.

The PPI network (https://string-db.org/) was mapped. Choose multiple proteins-gene symbol, and the minimum required interaction score was 0.400.

**Figure 1:**
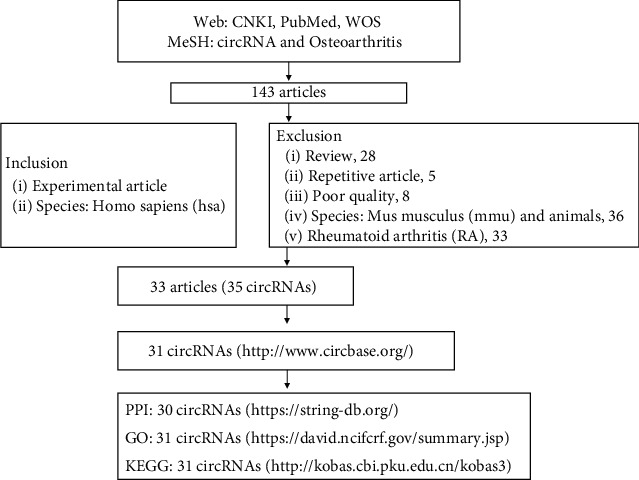
Flowchart.

GO analysis (DAVID, https://david.ncifcrf.gov/summary.jsp) and KEGG analysis (KOBAS, http://kobas.cbi.pku.edu.cn/kobas3) were performed to discuss the potential functions and the participating signaling pathways (species, Homo sapiens; input type, gene symbol; *p* < 0.05). As shown in Figure 1.

### 2.4. Data Processing and Analysis

The acquired data were analyzed and mapped using Cytoscape (3.7.2) and R language (R x64 4.0.2).

## 3. Results

### 3.1. Current Studies on the Correlation between circRNAs and OA

**Figure 2:**
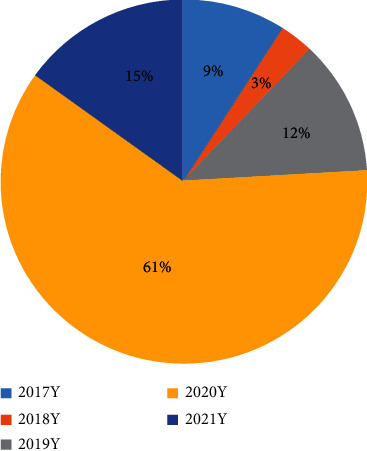
Year of issue of the 33 articles.

A total of 33 studies meeting the inclusion criteria were included in this study, and 35 circRNAs were sorted out by circRNA ID, gene symbol, regulation, miRNA, target gene/signal pathway, reference, and year (Table 1). As shown in Table 1, 81.82% of the articles about circRNAs in the recent 5 years discussed the circ-miRNA axis. As shown in Table 2, 27 circRNAs had upregulated expression and 8 had downregulated expression. As shown in Figure 2, most articles (61%) about circRNAs were published in 2020, and 15% of the articles were published in 2021.

### 3.2. PPI, GO, and KEGG Analyses of circRNAs

Repeated circRNAs (SERPINE2, VWF, EPS15, and UNK) and those having no information in circBase (hsa_circ_9119, hsa_circ_7, *PSM3*, and hsa_circ_100226) were excluded, and a total of 31 circRNAs were finally included in the PPI, GO, and KEGG analyses. The final result showed *p* < 0.05.

**Figure 3:**
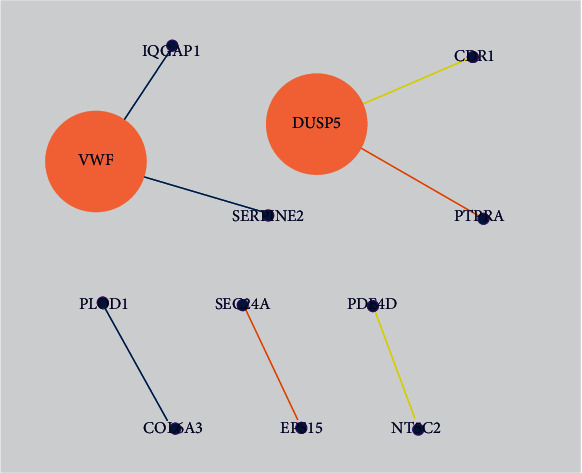
PPI network of circRNAs. Note: map node size to degree and map edge size to combined score, low values to small sizes and bright colors.

Figure 3 of PPI analysis shows a network of 30 circRNAs (RP11-909M7.3 not found in STRING); of these, 12 map nodes and 7 map edges were interrelated. VWF and DUSP5 had the biggest map node size (degree 2); IQGAP1-VWF-SERPINE2 and PLOD1-COL6A3 had the bigger map edge size (0.906, 0.928, and 0.913).

**Figure 4:**
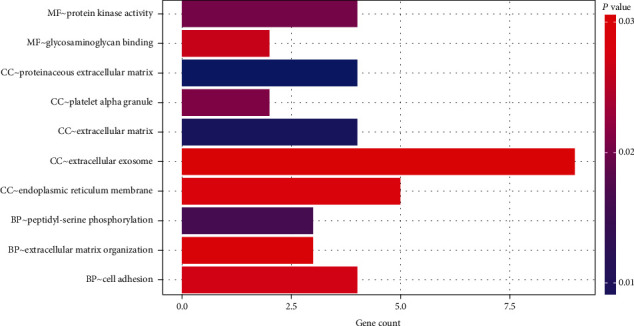
GO analysis of the 31 circRNAs.

GO enrichment analysis usually covers molecular function (MF), cellular component (CC), and biological process (BP). The results of this study showed that the functions of 31 circRNAs were mainly focused on MF, including protein kinase activity and glycosaminoglycan binding; CC were proteinaceous ECM, platelet *α* granule, extracellular matrix (ECM), cellular exosome, and endoplasmic reticulum membrane; BP included peptidyl-serine phosphorylation, ECM organization, and cell adhesion (Figure 4). Among them, VWF showed a majority of the functions (6/10, Table 3).

**Figure 5:**
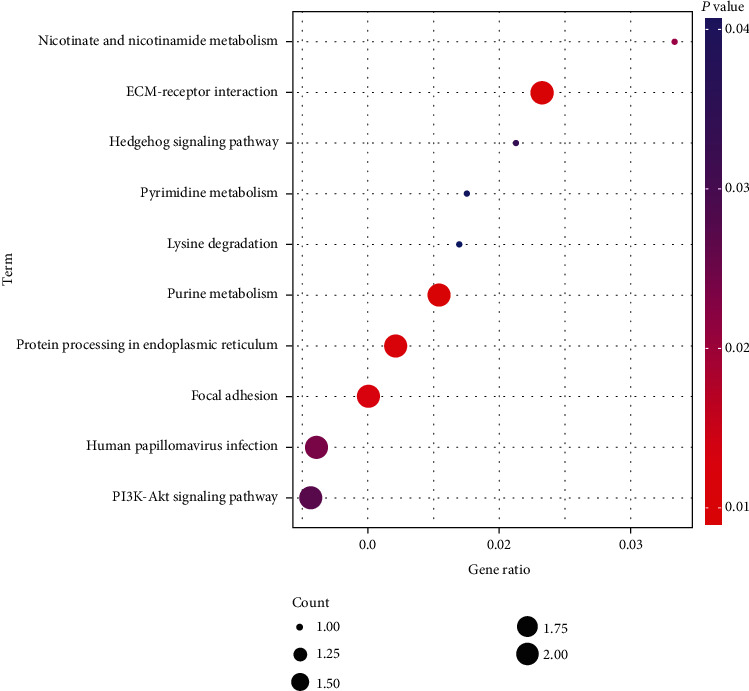
KEGG analysis of the 31 circRNAs.

KEGG signaling pathway analysis showed that circRNAs are enriched in PI3K/AKT, human papillomavirus infection (HPI), focal adhesion (FA), and other seven pathways (Figure 5), and VWF and COL6A3 were involved in 4/7 pathways (Table 4).

## 4. Discussion

### 4.1. Brief Information and Functions of circRNAs

circRNAs are a type of noncoding RNAs mainly found in the cytoplasm of mammalian cells. circRNAs consist of the 3′- and 5′-phosphodiester bonds covalently linked to form a circular structure, which is stable and resistant to RNA exonuclease-mediated degradation, and hence are termed as circRNAs. Three types of circRNAs, exon circRNAs (exon circular RNA (ecircRNAs)), intron circRNAs (intron circular RNA (ciRNAs)), and exon-intron circRNAs (exon-intron circular RNA (EIcircRNAs)) [42–44], especially ciRNAs, are conserved across evolution and have a half-life of >48 h, which also confirms their high stability. In addition, circRNAs are highly stable and sensitive in body fluids and used for biochemical tests [45–47].

**Figure 6:**
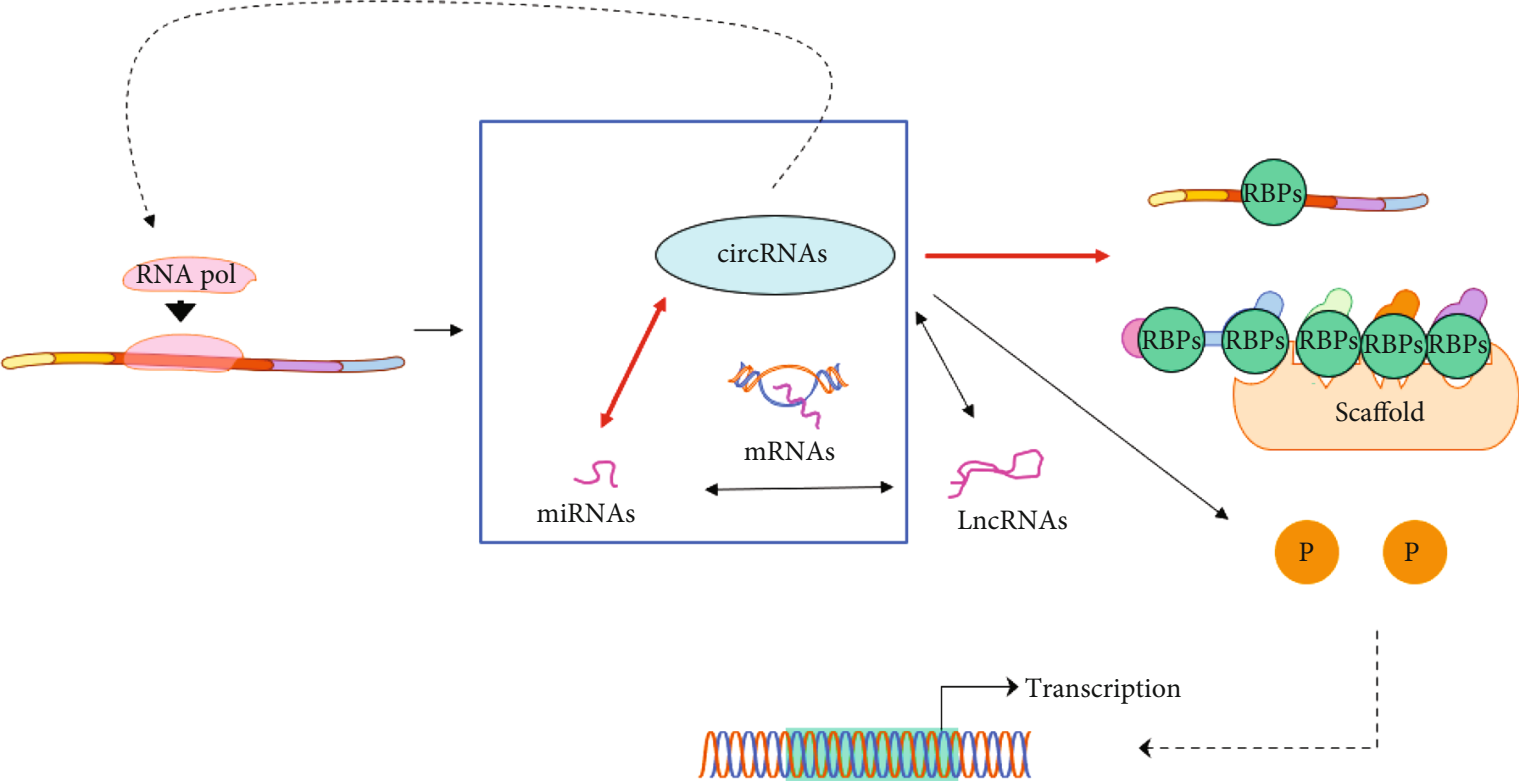
Functions of circRNAs.

Current studies showed that the functions of circRNAs are as follows. (1) They adsorb microRNAs (miRNAs), bind to miRNAs as sponges, affect the corresponding message RNAs (mRNAs), and eventually regulate the expression of target genes. (2) They regulate the activity of RNA-binding proteins (RBPs) and transport them or act as their scaffold to facilitate the formation of new complexes. Additionally, circRNAs can also interact with proteins, selectively cut or transcribe parent genes (binding RNA polymerases), and encode the proteins [48–52], as shown in Figure 6.

### 4.2. Studies on the Mechanism of circRNAs in OA and circRNAs as Biological Indicators of OA

This study showed that the articles on circRNAs in OA in the recent 5 years mainly focused on the mechanism while they also discussed circRNAs as clinical, biological indicators of OA.

According to the statistical results of this study, 61% of the relevant articles in the recent 5 years were published in 2020 and 15% in 2021. The majority of these articles focused on the mechanism of circ-miRNA with respect to the absorption effect of circRNAs as sponges on miRNAs in OA. Kulcheski et al. [53] proposed that circRNAs are sponges of miRNAs and can serve as the novel type of biomarkers. circRNA 0092516 regulates chondrocyte differentiation and apoptosis via miRNA-337-3p/PTEN (phosphatase and tensin homolog), according to Huang et al. [8], while circRNA UBE2G1 regulates lipopolysaccharide- (LPS-) induced OA chondrocytes via miR-373/hypoxic inducible factor 1 alpha (HIF-1*α*), according to Chen et al. [9]. Wu et al. [12] demonstrated that lowly expressed circRNA HIPK3 regulates SRY-related high-mobility group box gene 8 (SOX-8), a critical marker of chondrocyte development as the sponge of miR-124, thus promoting the apoptosis of osteoarthritis chondrocytes. Ma et al. [13] found that circRNA VCAN promotes the apoptosis of OA chondrocytes by blocking the NF-*κ*B signaling pathway. Wu et al. [16] showed that circRNA PDE4D protected OA by binding to miR-103a-3p and regulating the fibroblast growth factor 18 (FGF18), and Zhou et al. [17] found that circRNA ANKRD36 regulated *Casz1* (miR-599 target gene) and prevented the apoptosis and inflammation of OA chondrocytes by targeting miR-599.

Additionally, some studies also discussed circRNAs as biological indicators to detect and evaluate OA. In the study by Wang et al. [9], patients with Kashin–Beck disease (KBD) and OA were subjected to circRNA sequencing to observe differential expression; the result of which showed that circRNA 0020014 could serve as the potential marker of OA to evaluate the progression of OA. Wang et al. [23] analyzed the gene expression profile, wherein VWF (hsa_circ_0025119) and other three genes served as OA markers. Xiao et al. [26] demonstrated that, on the Illumina HiSeq platform, circRNA 0045714 was expressed differentially in OA. Xiang et al. [54] revealed the expression profile of circRNAs in OA through RNA sequencing and identified 122 circRNAs of differential expression. Based on these studies, VWF (hsa_circ_0025119) had the highest value (Figure 3, Tables 3 and 4), indicating a significant interaction between VWF and other circRNAs; also, additional functions and signaling pathways were detected in the BP. Therefore, we speculated that VWF (hsa_circ_0025119) is more feasible to be used as a biological indicator compared to other circRNAs, to detect OA in clinical practice.

### 4.3. Studies on the Potential Functions of circRNAs in OA and Involved Signaling Pathways

The current study showed that circRNAs play a critical role in ECM. Shen et al. [7] showed that the overexpression of circRNA SERPINE2 downregulates the miR-1271-ERG (E26 transformation-specific-related gene) pathway to reduce HCS (human chondrocyte) apoptosis and promote ECM anabolism, thus slowing down OA development. Zhu et al. [11] found that circRNA GCN1L1 regulates miR-330-3p and TNF-*α* to promote OA synovial cells and reduce ECM anabolism. Wu et al. [18] demonstrated that circRNA 0005105 upregulates the expression of NAMPT (miR-26a target gene) and promotes ECM degradation in chondrocytes by absorbing miR-26a as sponges. In addition, circRNA TMBIM6 promotes ECM degradation of OA-induced chondrocytes via the miR-27a/matrix metalloproteinase-13 (MMP-13) axis, according to Bai et al. [21]. circRNA SERPINE2 reduces IL-1*β*-induced apoptosis and ECM degradation of chondrocytes by regulating the miR-495/transforming growth factor-beta receptor 2 (TGFBR2) axis [32]. Furthermore, the functions of circRNAs also include protein kinase activity, glycosaminoglycan binding, endoplasmic reticulum membrane, and peptidyl-serine phosphorylation, which can be the focus of future studies on the mechanism of OA.

**Figure 7:**
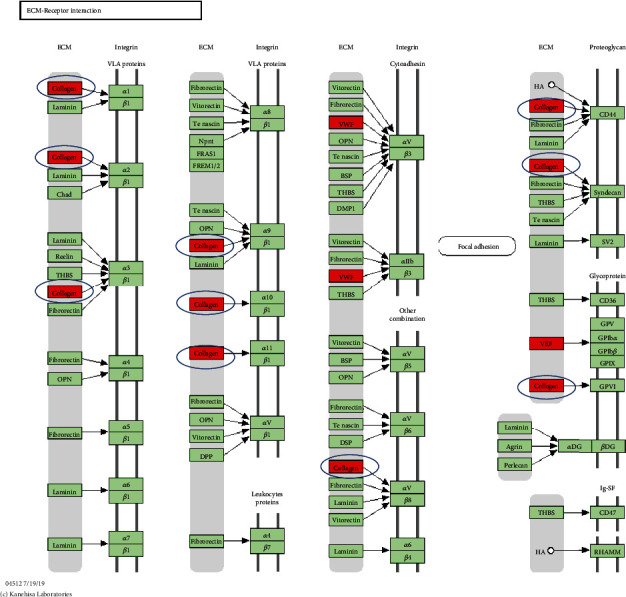
ECM-receptor interaction signaling pathway.

In this study, VWF and COL6A3 are involved in the PI3K/AKT signaling pathway (Table 4). According to Zhou et al. [24], circRNA7 regulates PI3K/AKT/mTOR by absorbing miR-7, thus aggravating OA and indicating that the PI3K/AKT signaling pathway may play a critical role in circRNAs regulating the development of OA. The PI3K/AKT/mTOR signaling pathway functions in cartilage degeneration, subchondral bone dysfunction, and synovial inflammation [55–57]. Therefore, in future studies on the mechanism of circRNA-regulated OA chondrocytes and synovial cells, the correlation between the circ-PI3K/AKT/mTOR axes can be observed, and the role of PI3K/AKT/mTOR is discussed. Multiple collagen factors were also detected in the ECM-receptor interaction pathway in Figure 5. Collagen is a vital component of cartilage composition and plays a crucial role in protecting cartilage tissues [58–60]. This finding suggested that the ECM-receptor interaction signaling pathway may also play a critical role in the mechanism underlying circRNA-regulated OA (Figure 7).

We also found that the expression of most circRNAs was upregulated, while a few were downregulated in OA. According to Wang et al. [31], circRNA RNF121 aggravated the progression of OA via the miR-665/MYD88 axis (MYD88 is the canonical adaptor for inflammatory pathway), and according to Xiao et al. [33], circRNA CSNK1G1 promotes the progression of OAs by targeting the miR-4428/FUT2 (fucosyltransferase) axis. Jiang et al. [35] demonstrated that circRNA DHRS3 accelerates OA progression via miR-183-5p/GREM1 (*Gremlin*, the miR-183-5p target gene). Wang et al. [38] found that circRNA 0114876 aggravates OA via the miR-671/TRAF2 (TNF receptor-associated factor 2) axis. Yang et al. [40] found that circRNA RSU1 aggravates OA via the miR-93-5p/MAP3K8 (mitogen-activated protein kinase 8) axis, and Shen et al. [14] showed that circRNA CDK14 protects OA via the sponge tissue miR-125a-5p and enhances the expression of *Smad2* (gene of TGF-*β* family). Moreover, in the study by Chen et al. [22], circRNA 9119 was shown to prevent apoptosis of IL-1*β*-treated OA chondrocytes by blocking the miR-26a/PTEN axis, and circRNA ADAMTS6 protects OA by absorbing miR-431-5p [36]. Another study showed that circRNA 0045714 exerted a protective effect on OA via the miR-193b/insulin-like growth factor 1 receptor (IGF1R) axis [37]. In summary, 77.78% of the circRNAs were upregulated and 22.23% were downregulated, and the overexpression of the majority of the circRNAs aggravates the occurrence and development of OA.

Herein, the studies on the correlation between circRNAs and OA in the recent 5 years and the circRNAs with differential expression and reliable mechanism of action in OA were reviewed. We found that most articles about circRNAs regulating OA in the recent 5 years focused on the mechanism, especially the absorption effect of circ-miRNA as sponges in the recent 2 years, while most of the articles about their functions addressed ECM and PI3K, AKT, and mTOR signaling pathways. Based on the GO and KEGG analysis results, future studies might focus on the functions of circRNAs, such as protein kinase activity, glycosaminoglycan binding, endoplasmic reticulum membrane, and peptidyl-serine phosphorylation, as well as ECM-receptor interaction-related signaling pathways. circRNA VWF, with preferable functions, interactions, and involvement, can be used as a biological indicator to detect OA in clinical practice. However, although the absorption effect of circ-miRNA as sponges in the mechanism of OA has been under intensive focus in the recent 2 years, studies are still rare. Therefore, further studies would focus on the database of the circ-miRNA axis in OA in order to provide a reference for the clinical treatment based on the mechanism of OA.

## Figures and Tables

**Table 1:** Articles about the correlation between circRNAs and OA in the recent 5 years.

	circRNA ID	Gene symbol	Regulation	miRNAs	Target gene/pathway	Reference	Year
1	hsa_circ_0141827	*SERPINE2*	Down	miR-1271	ERG pathway, SOX, COL2	Shen et al. [7]	2019
2	hsa_circ_0092516	*NT5C2*	Down	miR-337-3p	MMP-1, COL2	Huang et al. [8]	2020
3	hsa_circ_0020014	*DUSP5*	NA	NA	NA	Wang et al. [9]	2020
4	hsa_circ_0041552	*UBE2G1*	Down	miR-373	HIF-1a	Chen et al. [10]	2020
5	hsa_circ_0000448	*GCN1L1*	Down	miR-330-3p	TNF-*α*, ADAMTS4	Zhu et al. [11]	2020
6	hsa_circ_0021592	*HIPK3*	Down	miR-124	SOX-8	Wu et al. [12]	2020
7	hsa_circ_0129854	*VCAN*	NA	NA	NF-*κ*B pathway	Ma et al. [13]	2020
8	hsa_circ_0080978	*CDK14*	Down	miR-125a-5p	SOX-9, Smad-2	Shen et al. [14]	2020
9	hsa_circ_0136474	*ASH2L*	Down	miR-127-5p	MMP-13	Li et al. [15]	2019
10	hsa_circ_0129214	*PDE4D*	Down	miR-103a-3p	FGF18	Wu et al. [16]	2021
11	hsa_circ_0055722	*ANKRD36*	Down	miR-599	Casz1	Zhou et al. [17]	2021
12	hsa_circ_0005105	*SEC24A*	Down	miR-26a	NAMPT	Wu et al. [18]	2017
13	hsa_circ_0032131	*PRKCH*	Down	miR-1182	NA	Wang et al. [19, 20]	2019
14	hsa_circ_0026176	*TMBIM6*	Down	miR-27a	MMP-13	Bai et al. [21]	2020
15	hsa_circ_9119^#^	NA	Down	miR-26a	PTEN	Chen et al. [22]	2020
16	hsa_circ_0025119 hsa_circ_0025113 hsa_circ_0009897 hsa_circ_0002447	*VWF* *VWF* *PLOD1* *COL6A3*	NA	NA	NA	Wang et al. [23]	2020
17	hsa_circ_7^#^	NA	Down	miR-7	PI3K/AKT/mTOR	Zhou et al. [24, 25]	2020
18	hsa_circ_0045714 hsa_circ_0002485 hsa_circ_0005567	*UNK* *ATP9B* *EPS15*	NA	NA	NA	Xiao et al. [26]	2019
19	NA^#^	*PSM3*	Down	miR-296-5p		Ni et al. [27]	2020
20	hsa_circ_100226^#^	*MSR*	Down	miR-875	TNF-*α*	Liu et al. [28]	2017
21	hsa_circ_0001946	*CDR1*	Down	miR-641	COL2, IL-6	Zhang et al. [29]	2020
22	hsa_circ_0040639	*CDH13*	Down	miR-296-3p	PTEN	Zhou et al. [30]	2020
23	hsa_circ_0023404	*RNF121*	Down	miR-665	MYD88	Wang et al. [31]	2020
24	hsa_circ_0141827	*SERPINE2*	Down	miR-495	TGFBR2	Zhang et al. [32]	2020
25	hsa_circ_0035826	*CSNK1G1*	Down	miR-4428	FUT2	Xiao et al. [33]	2020
26	hsa_circ_0005567	*EPS15*	Down	miR-495	ATG14	Zhang et al. [34]	2020
27	hsa_circ_0010014	*DHRS3*	Down	miR-183-5p	GREM1	Jiang et al. [35]	2020
28	hsa_circ_0072655	*ADAMTS6*	Down	miR-431-5p	IL-*β*	Fu et al. [36]	2020
29	hsa_circ_0045714	*UNK*	Down	miR-193b	IGF1R	Li et al. [37]	2017
30	hsa_circ_0114876	*PTPRA*	Down	miR-671	TRAF2	Wang et al. [38]	2021
31	hsa_circ_0104873 hsa_circ_0104595 hsa_circ_0101251	*IQGAP1* *SCAPER* *RP11-909M7.3*	NA	NA	NA	Yu et al. [39]	2018
32	hsa_circ_0017855	*RSU1*	Down	miR-93-5p	MAP3K8	Yang et al. [40]	2021
33	hsa_circ_0045714	*UNK*	Down	miR-218-5p	HRAS	Jiang et al. [41]	2021

Note: “NA” means not available, and “#” means no relevant information in circBase.

**Table 2:** Expression of circRNAs in OA.

Expression of 35 circRNAs in OA	
Upregulation (27)	Downregulation (8)
NT5C2 [8], DUSP5 [9], UBE2G1 [10], GCN1L1 [11], HIPK3 [12], VCAN [13], ASH2L [15], SEC24A [18], PRKCH [19, 20], TMBIM6 [21], VWF [23], PLOD1 [23], COL6A3 [23], hsa_circ_7 [24, 25], ATP9B [26], PSM3 [27], MSR [28], CDR1 [29], CDH13 [30], RNF121 [31], CSNK1G1 [33], DHRS3 [35], PTPRA [38], IQGAP1 [39], SCAPER [39], RP11-909M7.3 ^[39]^, RSU1 [40]	SERPINE2 [7, 32], CDK14 [14], PDE4D [16], ANKRD36 [17], hsa_circ_9119 [22], EPS15 [26, 34], ADAMTS6 [36], UNK [37, 41]

**Table 3:** Functions of circRNAs.

Term	Genes
MF: protein kinase activity	*PRKCH*, *CDK14*, *HIPK3*, *CSNK1G1*
MF: glycosaminoglycan binding	*VCAN*, *SERPINE2*
CC: proteinaceous *COL6A3* extracellular matrix	*VCAN*, *VWF*, *COL6A3*, *ADAMTS6*
CC: platelet alpha granule	*SERPINE2*, *VWF*
CC: extracellular matrix	*VCAN*, *SERPINE2*, *VWF*
CC: extracellular exosome	*PRKCH*, *VWF*, *PTPRA*, *CDH13*, *COL6A3*, *UBE2G1*, *PLOD1*, *IQGAP1*, *RSU1*
CC: endoplasmic reticulum membrane	*SEC24A*, *TMBIM6*, *PLOD1*, *DHRS3*, *RNF121*
BP: peptidyl-serine phosphorylation	*PRKCH*, *HIPK3*, *CSNK1G1*
BP: extracellular matrix organization	*VCAN*, *VWF*, *COL6A3*
BP: cell adhesion	*VCAN*, *VWF*, *CDH13*, *COL6A3*

**Table 4:** Signaling pathways involving circRNAs.

Term	Input
ECM-receptor interaction	VWF|COL6A3
Purine metabolism	PDE4D|NT5C2
Protein processing in the endoplasmic reticulum	SEC24A|UBE2G1
Focal adhesion	VWF|COL6A3
Nicotinate and nicotinamide metabolism	NT5C2
Human papillomavirus infection	VWF|COL6A3
PI3K-Akt signaling pathway	VWF|COL6A3
Hedgehog signaling pathway	CSNK1G1
Pyrimidine metabolism	NT5C2
Lysine degradation	PLOD1

## References

[1] van den Bosch M. H. J. (2021). Osteoarthritis year in review 2020: biology. *Osteoarthritis and Cartilage*.

[2] Billesberger L. M., Fisher K. M., Qadri Y. J., Boortz-Marx R. L. (2020). Procedural treatments for knee osteoarthritis: a review of current injectable therapies. *Pain Research & Management*.

[3] Hunt M. A., Charlton J. M., Esculier J. F. (2020). Osteoarthritis year in review 2019: mechanics. *Osteoarthritis and Cartilage*.

[4] Abramoff B., Caldera F. E. (2020). Osteoarthritis: pathology, diagnosis, and treatment options. *The Medical Clinics of North America*.

[5] Yu C. X., Sun S. (2018). An emerging role for circular RNAs in osteoarthritis. *Yonsei Medical Journal*.

[6] Li H. Z., Lin Z., Xu X. H., Lin N., Lu H. D. (2018). The potential roles of circRNAs in osteoarthritis: a coming journey to find a treasure. *Bioscience Reports*.

[7] Shen S., Wu Y., Chen J. (2019). CircSERPINE2 protects against osteoarthritis by targeting miR-1271 and ETS-related gene. *Annals of the Rheumatic Diseases*.

[8] Huang Z., Ma W., Xiao J., Dai X., Ling W. (2021). CircRNA_0092516 regulates chondrocyte proliferation and apoptosis in osteoarthritis through the miR-337-3p/PTEN axis. *The Journal of Biochemistry*.

[9] Wang Y., Wu C., Zhang Y. (2020). Screening for differentially expressed circRNA between Kashin-Beck disease and osteoarthritis patients based on circRNA chips. *Clinica Chimica Acta*.

[10] Chen G., Liu T., Yu B., Wang B., Peng Q. (2020). CircRNA-UBE2G1 regulates LPS-induced osteoarthritis through miR-373/HIF-1a axis. *Cell Cycle*.

[11] Zhu H., Hu Y., Wang C., Zhang X., He D. (2020). CircGCN1L1 promotes synoviocyte proliferation and chondrocyte apoptosis by targeting miR-330-3p and TNF-*α* in TMJ osteoarthritis. *Cell Death & Disease*.

[12] Wu Q., Yuan Z. H., Ma X. B., Tang X. H. (2020). Low expression of CircRNA HIPK3 promotes osteoarthritis chondrocyte apoptosis by serving as a sponge of miR-124 to regulate SOX8. *European Review for Medical and Pharmacological Sciences*.

[13] Ma H. R., Mu W. B., Zhang K. Y., Zhou H. K., Jiang R. D., Cao L. (2020). CircVCAN regulates the proliferation and apoptosis of osteoarthritis chondrocyte through NF-*κ*B signaling pathway. *European Review for Medical and Pharmacological Sciences*.

[14] Shen P., Yang Y., Liu G. (2020). CircCDK14 protects against osteoarthritis by sponging miR-125a-5p and promoting the expression of Smad2. *Theranostics.*.

[15] Li Z., Yuan B., Pei Z. (2019). Circ_0136474 and MMP-13 suppressed cell proliferation by competitive binding to miR-127-5p in osteoarthritis. *Journal of Cellular and Molecular Medicine*.

[16] Wu Y., Hong Z., Xu W. (2021). Circular RNA circPDE4D protects against osteoarthritis by binding to miR-103a-3p and regulating FGF18. *Molecular Therapy*.

[17] Zhou J. L., Deng S., Fang H. S., du X. J., Peng H., Hu Q. J. (2021). Circular RNA circANKRD36 regulates Casz1 by targeting miR-599 to prevent osteoarthritis chondrocyte apoptosis and inflammation. *Journal of Cellular and Molecular Medicine*.

[18] Wu Y., Zhang Y., Zhang Y., Wang J. J. (2017). CircRNA hsa_circ_0005105 upregulates NAMPT expression and promotes chondrocyte extracellular matrix degradation by sponging miR-26a. *Cell Biology International*.

[19] Wang Y., Wu C., Zhang F. (2019). Screening for differentially expressed circular RNAs in the cartilage of osteoarthritis patients for their diagnostic value. *Genetic Testing and Molecular Biomarkers*.

[20] Wang Y., Wu C., Yang Y., Ren Z., Lammi M. J., Guo X. (2019). Preliminary exploration of hsa_circ_0032131 levels in peripheral blood as a potential diagnostic biomarker of osteoarthritis. *Genetic Testing and Molecular Biomarkers*.

[21] Bai Z. M., Kang M. M., Zhou X. F., Wang D. (2020). CircTMBIM6 promotes osteoarthritis-induced chondrocyte extracellular matrix degradation via miR-27a/MMP13 axis. *European Review for Medical and Pharmacological Sciences*.

[22] Chen C., Yin P., Hu S., Sun X., Li B. (2020). Circular RNA-9119 protects IL-1*β*-treated chondrocytes from apoptosis in an osteoarthritis cell model by intercepting the microRNA-26a/PTEN axis. *Life Sciences*.

[23] Wang B., Zhong J. L., Xu X. H. (2021). Gene expression profiling analysis to identify key genes and underlying mechanisms in meniscus of osteoarthritis patients. *Combinatorial Chemistry & High Throughput Screening*.

[24] Zhou X., Li J., Zhou Y. (2020). Down-regulated ciRS-7/up-regulated miR-7 axis aggravated cartilage degradation and autophagy defection by PI3K/AKT/mTOR activation mediated by IL-17A in osteoarthritis. *Aging*.

[25] Zhou X., Jiang L., Fan G. (2019). Role of the ciRS-7/miR-7 axis in the regulation of proliferation, apoptosis and inflammation of chondrocytes induced by IL-1*β*. *International Immunopharmacology*.

[26] Xiao K., Xia Z., Feng B. (2019). Circular RNA expression profile of knee condyle in osteoarthritis by illumina HiSeq platform. *Journal of Cellular Biochemistry*.

[27] Ni J. L., Dang X. Q., Shi Z. B. (2020). CircPSM3 inhibits the proliferation and differentiation of OA chondrocytes by targeting miRNA-296-5p. *European Review for Medical and Pharmacological Sciences*.

[28] Liu Q., Zhang X., Hu X. (2017). Emerging roles of circRNA related to the mechanical stress in human cartilage degradation of osteoarthritis. *Molecular Therapy - Nucleic Acids*.

[29] Zhang W., Zhang C., Hu C., Luo C., Zhong B., Yu X. (2020). Circular RNA-CDR1as acts as the sponge of microRNA-641 to promote osteoarthritis progression. *Journal of Inflammation*.

[30] Zhou Z., Ma J., Lu J., Chen A., Zhu L. (2021). Circular RNA CircCDH13 contributes to the pathogenesis of osteoarthritis via CircCDH13/miR-296-3p/PTEN axis. *Journal of Cellular Physiology*.

[31] Wang T., Hao Z., Liu C. (2020). LEF1 mediates osteoarthritis progression through circRNF121/miR-665/MYD88 axis via NF-*к*B signaling pathway. *Cell Death & Disease*.

[32] Zhang Q., Qiao X., Xia W. (2020). CircSERPINE2 weakens IL-1*β*-caused apoptosis and extracellular matrix degradation of chondrocytes by regulating miR-495/TGFBR2 axis. *Bioscience Reports*.

[33] Xiao J., Wang R., Zhou W., Cai X., Ye Z. (2020). Circular RNA CSNK1G1 promotes the progression of osteoarthritis by targeting the miR‑4428/FUT2 axis. *International Journal of Molecular Medicine*.

[34] Zhang J., Cheng F., Rong G., Tang Z., Gui B. (2020). Hsa_circ_0005567 activates autophagy and suppresses IL-1*β*-induced chondrocyte apoptosis by regulating miR-495. *Frontiers in Molecular Biosciences*.

[35] Jiang R., Gao H., Cong F., Zhang W., Song T., Yu Z. (2020). Circ_DHRS3 positively regulates GREM1 expression by competitively targeting miR-183-5p to modulate IL-1*β*-administered chondrocyte proliferation, apoptosis and ECM degradation. *International Immunopharmacology*.

[36] Fu Q., Li L., Wang B. (2021). CircADAMTS6/miR-431-5p axis regulate interleukin-1*β* induced chondrocyte apoptosis. *The Journal of Gene Medicine*.

[37] Li B. F., Zhang Y., Xiao J. (2017). Hsa_circ_0045714 regulates chondrocyte proliferation, apoptosis and extracellular matrix synthesis by promoting the expression of miR-193b target gene IGF1R. *Human Cell*.

[38] Wang Q., Luo S., Yang J. (2021). Circ_0114876 promoted IL-1*β*-induced chondrocyte injury by targeting miR-671/TRAF2 axis. *Biotechnology Letters*.

[39] Yu F., Xie C., Sun J., Feng H., Huang X. (2018). Circular RNA expression profiles in synovial fluid: a promising new class of diagnostic biomarkers for osteoarthritis. *International Journal of Clinical and Experimental Pathology*.

[40] Yang Y., Shen P., Yao T. (2021). Novel role of circRSU1 in the progression of osteoarthritis by adjusting oxidative stress. *Theranostics*.

[41] Jiang H., Dai J., Zhang C., Sun H., Tang X. (2021). Circ_0045714 alleviates TNF-*α*-induced chondrocyte injury and extracellular matrix degradation through miR-218-5p/HRAS axis. *Journal of Bioenergetics and Biomembranes*.

[42] Li R., Jiang J., Shi H., Qian H., Zhang X., Xu W. (2020). CircRNA: a rising star in gastric cancer. *Cellular and Molecular Life Sciences*.

[43] Zang J., Lu D., Xu A. (2020). The interaction of circRNAs and RNA binding proteins: an important part of circRNA maintenance and function. *Journal of Neuroscience Research*.

[44] Shi Y., Jia X., Xu J. (2020). The new function of circRNA: translation. *Clinical & Translational Oncology*.

[45] Wesselhoeft R. A., Kowalski P. S., Anderson D. G. (2018). Engineering circular RNA for potent and stable translation in eukaryotic cells. *Nature Communications*.

[46] Chen L. L. (2020). The expanding regulatory mechanisms and cellular functions of circular RNAs. *Nature Reviews Molecular Cell Biology*.

[47] Shi X., Wang B., Feng X., Xu Y., Lu K., Sun M. (2020). circRNAs and exosomes: a mysterious frontier for human cancer. *Molecular Therapy - Nucleic Acids*.

[48] Huang A., Zheng H., Wu Z., Chen M., Huang Y. (2020). Circular RNA-protein interactions: functions, mechanisms, and identification. *Theranostics*.

[49] Xiao M. S., Ai Y., Wilusz J. E. (2020). Biogenesis and functions of circular RNAs come into focus. *Trends in Cell Biology*.

[50] Zhou W. Y., Cai Z. R., Liu J., Wang D. S., Ju H. Q., Xu R. H. (2020). Circular RNA: metabolism, functions and interactions with proteins. *Molecular Cancer*.

[51] Yang Q., Li F., He A., Yang B. B. (2021). Circular RNAs: expression, localization, and therapeutic potentials. *Molecular Therapy*.

[52] Zhang W., Qi L., Chen R. (2021). Circular RNAs in osteoarthritis: indispensable regulators and novel strategies in clinical implications. *Arthritis Research & Therapy*.

[53] Kulcheski F. R., Christoff A. P., Margis R. (2016). Circular RNAs are miRNA sponges and can be used as a new class of biomarker. *Journal of Biotechnology*.

[54] Xiang S., Li Z., Bian Y., Weng X. (2019). RNA sequencing reveals the circular RNA expression profiles of osteoarthritic synovium. *Journal of Cellular Biochemistry*.

[55] Sun K., Luo J., Guo J., Yao X., Jing X., Guo F. (2020). The PI3K/AKT/mTOR signaling pathway in osteoarthritis: a narrative review. *Osteoarthritis and Cartilage*.

[56] Zhang Y., Cai W., Han G. (2020). Panax notoginseng saponins prevent senescence and inhibit apoptosis by regulating the PI3K‑AKT‑mTOR pathway in osteoarthritic chondrocytes. *International Journal of Molecular Medicine*.

[57] Xue J. F., Shi Z. M., Zou J., Li X. L. (2017). Inhibition of PI3K/AKT/mTOR signaling pathway promotes autophagy of articular chondrocytes and attenuates inflammatory response in rats with osteoarthritis. *Biomedicine & Pharmacotherapy*.

[58] Honvo G., Lengelé L., Charles A., Reginster J. Y., Bruyère O. (2020). Role of collagen derivatives in osteoarthritis and cartilage repair: a systematic scoping review with evidence mapping. *Rheumatology and Therapy*.

[59] Etich J., Rehberg M., Eckes B., Sengle G., Semler O., Zaucke F. (2020). Signaling pathways affected by mutations causing osteogenesis imperfecta. *Cellular Signalling*.

[60] Gencoglu H., Orhan C., Sahin E., Sahin K. (2020). Undenatured type II collagen (UC-II) in joint health and disease: a review on the current knowledge of companion animals. *Animals*.

